# Optimization of methods for intrasplenic administration of human amniotic epithelial cells in order to perform safe and effective cell-based therapy for liver diseases

**DOI:** 10.1007/s12015-024-10735-1

**Published:** 2024-05-21

**Authors:** Piotr Czekaj, Mateusz Król, Emanuel Kolanko, Patrycja Wieczorek, Edyta Bogunia, Mateusz Hermyt, Aniela Grajoszek, Agnieszka Prusek

**Affiliations:** 1 Department of Cytophysiology, Chair of Histology and Embryology, Faculty of Medical Sciences in Katowice, Medical University of Silesia in Katowice, Medyków 18, Katowice, 40-752 Poland; 2 Department of Experimental Medicine, Medical University of Silesia in Katowice, Medyków 4, Katowice, 40-752 Poland

**Keywords:** Human amniotic epithelial cells, Cell transplantation, Intrasplenic administration, Splenic port, Cell-based therapy, Liver failure

## Abstract

In animal experimental models the administration of stem cells into the spleen should ensure high effectiveness of their implantation in the liver due to a direct vascular connection between the two organs. The aim of this study was to update the methods of experimental intrasplenic cell transplantation using human amniotic epithelial cells (hAECs) which are promising cells in the treatment of liver diseases. BALB/c mice were administered intrasplenically with 0.5, 1, and 2 million hAECs by direct bolus injection (400 µl/min) and via a subcutaneous splenic port by fast (20 μl/min) and slow (10 μl/min) infusion. The port was prepared by translocating the spleen to the skin pocket. The spleen, liver, and lungs were collected at 3 h, 6 h, and 24 h after the administration of cells. The distribution of hAECs, histopathological changes in the organs, complete blood count, and biochemical markers of liver damage were assessed. It has been shown that the method of intrasplenic cell administration affects the degree of liver damage. The largest number of mice showing significant liver damage was observed after direct administration and the lowest after slow administration through a port. Liver damage increased with the number of administered cells, which, paradoxically, resulted in increased liver colonization efficiency. It was concluded that the administration of 1 × 10^6^ hAECs by slow infusion via a subcutaneous splenic port reduces the incidence of complications at the expense of a slight decrease in the effectiveness of implantation of the transplanted cells in the liver.

## Introduction

The therapeutic method used in end-stage liver disease in the course of acute or chronic liver damage is liver transplantation. In recent years, it has been possible to support this therapy with transplantation of hepatocytes alone, which may extend the time to liver transplantation or help rebuild organ reserves [1]. Unfortunately, due to the difficulties associated with the isolation of hepatocytes of appropriate quality, cell therapies with their application in liver diseases are still imperfect from the point of view of the achieved clinical effects [2].

An alternative to hepatocyte transplantation or an adjuvant therapy in the treatment of liver diseases could be stem cells, including cells isolated from human amniotic epithelium (hAECs). A major advantage of these cells, resulting from their function in the placenta, is their possession of strong immunomodulatory properties and characteristics of pluripotent cells [3]. Owing to their immune privilege properties, including induction of apoptosis of activated T cells, inhibition of T cell proliferation, and suppression of activated NK cells [4], hAECs are well tolerated in fully immunocompetent animal models as xenografts [5].

To date, preclinical trials using mouse models of liver diseases in which hAEC injections were employed have shown their significant potential to support liver regeneration. In a model of phenylketonuria, phenylalanine levels in the brains of mice normalized after direct intrahepatic injection of hAECs [1]. In a model of maple syrup urine disease (MSUD), hAECs co-injected with hepatocytes contributed to equilibration of branched-chain amino acids (BCAA) in the blood and brain [6]. Therapeutic properties, particularly anti-fibrotic properties of intrasplenically administered hAECs, have also been demonstrated in a mouse model of D-galactosamine intoxication [1].

One of the fundamental problems to be solved when planning a study and analyzing the therapeutic effects of the cell therapy is the assessment of the distribution of the administered cells in connection with the technique of their administration and in the context of their therapeutic efficacy [7]. This indirectly relates to answering the question to what extend the therapeutic effect depends on implantation in the damaged organ and to what extent on the survival of a sufficiently large number of cells, not necessarily in the immediate vicinity of the damaged sites. In clinical trials using allogeneic hepatocytes, the most common route of cell administration is via the portal vein or, in children, via the umbilical vein [8, 9]. Due to difficult accessibility of the portal vein, the main routes of stem cell delivery in the mouse model are intravenous (into the tail vein) and intraperitoneal. However, the consequence of such administration is a multi-organ distribution of cells with reduced numbers in the desired organ [10].

In the liver disease models, effective cell distribution with a high proportion of the administered cells localized in the liver parenchyma seems to be crucial. For this reason, the best method of administering hAECs in liver diseases seems to be direct injection into the liver, although it is associated with a high perioperative risk, as the administered cells may enter the hepatic venous system and obtaining hemostasis can be difficult too [11]. The route of hAEC administration with a lower risk of bleeding and ensuring effective implantation of cells in the liver is direct intrasplenic injection. The expected high rate of cell distribution in the liver is due to vascular connection of the spleen and the liver via the portal vein. Unfortunately, the administration of cells in this manner is highly invasive and requires deep anesthesia. Moreover, animals after this surgical procedure require a few days of postoperative recovery. This prevents multiple administration of cells in short intervals. One potential solution to this problem is to create a subcutaneous splenic port [12].

A subcutaneous splenic port is prepared by extracting the spleen from the peritoneal cavity and placing it in a previously created skin pocket without disturbing the vessels. After the wound has healed, the spleen is clearly visible and palpable through the skin [12]. This enables the administration of cells by intrasplenic injection without the need to open the peritoneal cavity, and even multiple administrations are possible.

In addition to many advantages of the port technique, there are potential complications, generally associated with intrasplenic cell administration. One of the drawbacks to consider when administering hAECs in experimental models using small animals, especially rodents, either via a port or by direct injection, is the disproportion between the hAEC diameter and the width of the sinusoids in the liver. As a result, relatively large hAECs can form emboli in the sinusoids of the recipient, especially if administered in large numbers. This effect is known from experiments in which hAECs were administered into a peripheral vein and visualized in pulmonary capillary embolisms [13]. The rate of hAEC administration may also be important. Studies using a subcutaneous splenic port have shown that hAEC can be administered safely and effectively even multiple times, but the number and rate of hAEC administration has not yet been optimized. In this study, hAEC administration was optimized in terms of the technique used, duration of injection, and number of administered cells considering possible postoperative complications.

## Materials and Methods

### Animals

The study was performed on 84 six-week-old female BALB/c mice weighing 18–25 g, purchased from the Animal House of the Center for Experimental Medicine of the Medical University of Silesia in Katowice. During the experiment, mice were housed in cages (three per cage) under standard conditions of temperature (22 °C ± 2 °C), humidity (50–60%), light/dark cycle (12 h/12 h), and light intensity (60–400 lx) with ad libitum access to water and standard laboratory chow (Labofeed). Animals were not fasted during the experiment.

### Experimental design

The animals were divided into 27 subgroups differing in the technique of administration (direct and via the subcutaneous splenic port — fast and slow), number of administered cells (0.5, 1 and 2 million), and time from hAEC injection to the endpoint of the experiment (3 h, 6 h, and 24 h) (Table 1).

**Table 1:** Experimental design concerning the method of hAEC infusion (D, direct; F, fast; S, slow), number of injected cells (0.5, 1, 2 × 10^6^), and experimental time-points (3 h, 6 h, 24 h)

Method of cell delivery		Cell number	Experimental time points/subgroup names		
			3 h	6 h	24 h
Direct intrasplenic bolus injection (400 µl/min); Total duration: 35–40 s		0.5 × 10^6^	3-D-0.5	6-D-0.5	24-D-0.5
		1 × 10^6^	3-D-1	6-D-1	24-D-1
		2 × 10^6^	3-D-2	6-D-2	24-D-2
via the subcutaneous splenic port	Fast infusion using a syringe pump (20 μl/min with 10 min break); Total duration: 22.5 min	0.5 × 10^6^	3-F-0.5	6-F-0.5	24-F-0.5
		1 × 10^6^	3-F-1	6-F-1	24-F-1
		2 × 10^6^	3-F-2	6-F-2	24-F-2
	Slow infusion using a syringe pump (10 μl/min with 10 min break); Total duration: 35 min	0.5 × 10^6^	3-S-0.5	6-S-0.5	24-S-0.5
		1 × 10^6^	3-S-1	6-S-1	24-S-1
		2 × 10^6^	3-S-2	6-S-2	24-S-2

The subcutaneous splenic port enabled a minimally invasive and long-lasting cell infusion procedure. The procedure consisted of translocation of the spleen from the abdominal cavity to the previously created skin pocket while preserving vascular continuity. After preparation of the splenic port, the mice were cultured for 7 days prior to administration of the cells. In addition, control groups were created (*n* = 3), in which 250 µl of 0.9% NaCl was injected after 24 h. The control groups in this experiment were set up to determine blood biochemical parameters and assess morphological and histopathological changes in the organs of mice undergoing sham surgery (without cell administration).

At 3 h, 6 h, and 24 h after cell administration and in the control groups, the mice were euthanized and 1 mL of orbital sinus blood was collected. Liver function tests that measure alanine transaminase (ALT), aspartate transaminase (AST), alkaline phosphatase (ALP), glucose (Gluc), total protein (TP), total bilirubin (TB), and complete blood count were provided to assess liver damage. The liver, spleen, and lungs were collected for histopathology.

### Direct intrasplenic administration of hAECs

**Fig. 1:**
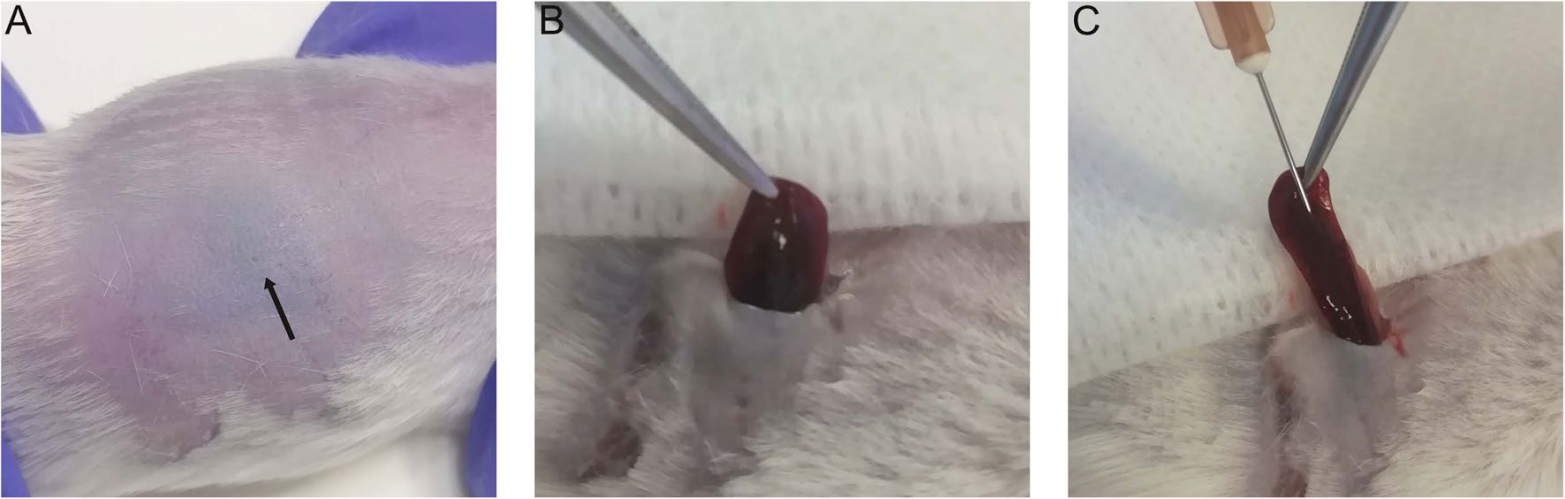
Direct intrasplenic direct injection of hAECs into mice: (**A**) spleen shadow (marked with an arrow) indicates the incision site, (**B**) spleen removal from the peritoneal cavity, and (**C**) hAEC injection

Tests of direct intrasplenic cell administration to anaesthetized mice involved visualization of the so-called spleen shadow (Fig. 1A), which enabled precise incision and removal of the organ from the body cavity (Fig. 1B). The spleen prepared in this way was injected with 0.5, 1, and 2 × 10^6^ of hAECs suspended in 250 μl of normal saline (Fig. 1C). After the procedure, the wound was carefully sutured and the mouse was placed in a separate cage.

### Preparation of a subcutaneous splenic port

**Fig. 2:**
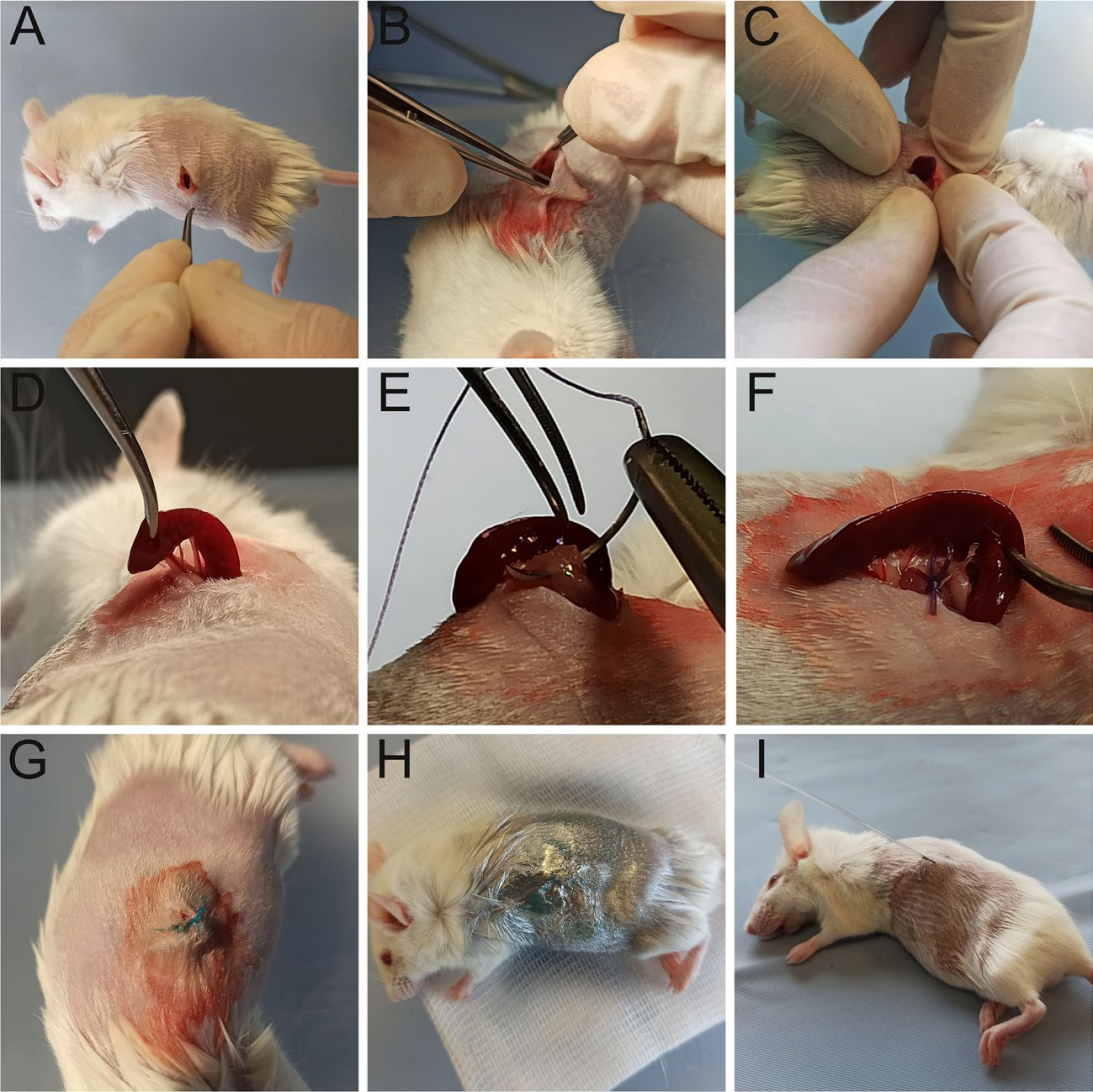
Construction of the subcutaneous splenic port: (**A**) preparation of the surgical field of the mouse and incision of the skin over the spleen, (**B**) creation of a subcutaneous pocket, (**C** and **D**) translocation of the spleen, (**E** and **F**) suturing the fascia at the avascular area of the splenic vascular pedicle, (**G**) suturing the skin and leaving the spleen in the subcutaneous pocket, (**H**) application of a dressing and housing the mouse for 7 days, and (**I**) administration of hAECs by syringe pump

The subcutaneous splenic port was prepared according to the protocol developed by Miki et al. [12]. First, hair was removed from the back of each mouse. The surgical field was then disinfected. A small incision was made in the vicinity of the spleen. The spleen was then removed and placed in a subcutaneous pocket. The muscle layer was subsequently closed over the avascular area between the splenic vessels to keep the spleen in the subcutaneous pocket. Finally, the skin was closed (Fig. 2).

### Isolation of epithelial cells from the amnion

Placentas of healthy women aged 25 to 40 were collected after obtaining informed consent from the patients. After separation from the chorion, the amniotic membrane was washed extensively in a sterile vessel containing fresh Plasmalyte solution to remove blood. The membrane was then divided into 2–3 g fragments, which were placed in 0.05% trypsin solution and incubated for 40 min at 37 °C in a shaking incubator (Enviro-Genie). Next, the membrane fragments were washed several times in fresh Plasmalyte solution. The hAEC suspensions obtained after washing the membrane were combined, a medium solution was added (DMEM, 20% FBS, 1% antibiotic, 1% L-glutamine), and the mixture was centrifuged (5 min, 500xg, at 4 °C). The supernatant was discarded and the resulting cell pellet was resuspended in 5 ml culture medium (DMEM, 1% L-glutamine, 10% FBS, 1% A/A, 10 ng/µl EGF).

The isolated cells were counted using a Moxi cell counter (Orflo). The effectiveness of hAEC isolation from the amniotic membrane of the examined placentas averaged 5 million cells/g of membrane. Three million cells were seeded into a 25 cm^3^ culture flask for cell count, viability, and phenotypic analysis, while the remaining cells were banked.

### Characterization of isolated cells

After 24 h of culture, the cells were digested with TrypLE, counted using a Moxi cell counter (Orflo), and labeled for flow cytometric assessment of viability and expression of markers of pluripotent cells (SSEA-4), epithelial cells (cytokeratins 14,15,16,19), and mesenchymal cells (CD73, CD105, CD44, CD90) as well as the immunomodulatory protein B7-H3.

To assess viability, cells were washed with PBS solution and then suspended in a labeling solution (Binding Buffer 1X; BD). Next, 5 µl of annexin V and 5 µl of propidium iodide were added to the cell suspension. After a 15-min incubation in the dark at room temperature, the numbers of viable, apoptotic, and dead cells in the isolated population were determined on a CytoFlex flow cytometer (Beckman Coulter).

To label the surface markers, the cells were washed with PBS solution, and then an appropriate amount was suspended in the labeling medium (PBS, 10% FBS, 1% EDTA). Specific antibodies were added to the samples thus prepared (Table 2).

**Table 2:** Antibodies and isotype controls used to assess the expression of cell markers by flow cytometry

Antibody	Manufacturer	Isotype control	Manufacturer
PE Mouse Anti-Human B7-H3 (no. FAB1027P)	R&D	PE Mouse IgG1, κ Isotype Control (no. IC002P)	R&D
FITC Mouse Anti-SSEA-4 (no. 560126)	Becton, Dickinson and Company	FITC Mouse IgG3, κ Isotype Control (no. 559806)	Becton, Dickinson and Company
PE Mouse Anti-Human CD73 (no. 550257)		PE Mouse IgG1, κ Isotype Control (no. 555749)	
APC Mouse Anti-Human CD105 (no. 562408)		APC Mouse IgG1, κ Isotype Control (no. 555751)	
FITC Mouse Anti-Human CD44 (no. 560977)		FITC Mouse IgG2b, κ Isotype Control (no. 555742)	
PE-Cy™7 Mouse Anti-Human CD90 (no. 561558)		PE-Cy™7 Mouse IgG1, κ Isotype Control (no. 557872)	
PE Mouse Anti-Human Cytokeratin 14, 15, 16, 19 Set (no. 550953)			

The samples were incubated with the antibody at room temperature for 30 min. in the absence of light, after which the cells were washed twice with PBS and suspended in 400 μl of labeling medium. Flow cytometric analysis was performed on a CytoFlex flow cytometer (Beckman Coulter). Compensation was performed using beads labeled with appropriate antibodies (VersaComp Antibody Capture Bead Kit). Non-antibody-labeled samples were used to eliminate cell autofluorescence.

### Preparation of hAECs for administration to animals

At 24 h before the scheduled administration, cells were seeded into a 75 cm^2^ culture flask (DMEM, 10% FBS, 1% AA, 1% L-glutamine, 10 ng/ml EGF). After this time, the cells were directed with TrypLE and counted using a Moxi cell counter (Orflo). To obtained a single cell suspension, a solution of DNase I was added (0.1 mg/mL) dissolved in CaCl_2_ and incubated at room temperature for 15 min. To inhibit the enzyme, culture medium with 2% FBS was added to the cells. The cell suspension was centrifuged (300xg, 10 min at room temperature). The supernatant was discarded, and the resulting pellet was suspended in the culture medium and passed through a 100-µm cell strainer. The cells were stored on ice until intrasplenic administration to mice. Just before administration, cells were centrifuged (5 min, 500xg, at 4 °C), and the resulting cell pellet was suspended in 250 µl of normal saline. Infusions of 0.5 million, 1 million, and 2 million hAECs at different flow rates were done according to the schedule presented in Table 1.

### Histopathological analysis of injury to the liver parenchyma

The liver samples were taken from the left lateral lobe, fixed in 10% buffered formaldehyde solution, processed by standard paraffin technique, and stained with hematoxylin and eosin (H&E). Interface hepatitis and parenchymal injury were assessed by a widely used simple grading and staging system [14–16] (Table 3). The necrosis grading algorithm was specifically designed for this study. The repeatability of the scoring method was assessed via evaluation of intra- and inter-observer correlation. Intra-observer repeatability (κ = 0.89), and inter-observer repeatability (κ = 0.76) were substantial.

**Table 3:** Scales for assessing liver damage based on markers related to inflammation, parenchymal injury, and extent of necrosis. These scales were used to create a semi-quantitative three-grade scale for histopathological assessment of the liver damage. Assessment of parenchymal injury included the presence of hepatocytic degeneration, apoptotic bodies, necrotic foci, significant hepatocyte edema, and inflammatory foci

Markers of liver injury		Basic markers of liver damage				
	Grade		Grade		Grade	
Interface hepatitis	1	Lack of inflammatory infiltration in periportal areas OR inflammatory infiltration in few areas	2	Inflammatory infiltration in most periportal areas. Infiltrate occupies less than < 50% of periportal areas circumference	3	Inflammatory infiltration in most periportal areas. Infiltrate occupies more than > 50% of periportal areas circumference
Parenchymal injury	1	Lack od parenchyma injury OR parenchymal oedema OR few lesions in whole specimen	2	At least one focus of parenchymal injury in field of view at magnification × 100	3	Necrosis of parenchyma OR numerous foci of parenchymal injury in field of view at magnification × 100 OR loss of physiological parenchymal architecture
Necrosis	1	Lack of liver necrosis	2	Few necrotic foci	3	Necrosis occupies areas around majority of periportal or pericentral areas

Both scoring algorithms were used in this study as the basis for the construction of a semi-quantitative three-grade scale (Table 4) according to previous general recommendations [17].

**Table 4:** A semi-quantitative three-grade scale for general histopathological assessment of the liver damage. The scale takes into account the assessment of the basic markers of liver damage (related to inflammation, parenchymal injury, and extent of necrosis) presented in Table 3

Grade	Assesment of the liver injury	Criteria for liver injury grading
I	Lack of histopathological changes in liver	1 point in every scale assesing basic markers of liver damage
II	Any pathological alternation detected in liver	> 1 point in at least one scale assesing basic markers of liver damage
III	Serious liver injury	3 points in at least one scale assesing basic markers of liver damage

### Immunohistochemical assessment of the distribution of transplanted hAECs

Paraffinized 4-µm-thick liver, lung, and spleen sections were dewaxed and rehydrated. Endogenous peroxidase activity was quenched with 0.6% H_2_O_2_ for 10 min. The sections were immunohistochemically stained to detect NuMA^+^ hAECs within tissues. Sections stained with isotype-matched mouse IgG served as negative controls. To visualize NuMA, antigens were retrieved by incubating with citric acid-based antigen unmasking solution (Vector Laboratories) for 60 min. Blocking of non-specific binding was done using 2.5% equine serum (Vector Laboratories) for 60 min. Subsequently, liver sections were incubated with rabbit anti-human NuMA antibody (ab84680; Abcam) diluted 1:1000 for 20 h at 4 °C. Next, sections were incubated with appropriate anti-rabbit secondary antibody conjugated with peroxidase (Vector Laboratories) at room temperature for 30 min. Immunoreactivity was visualized using 0.05% diaminobenzidine (Vector Laboratories). Sections taken from the human placenta served as a positive control. Each slide was photographed at 100 × magnification to cover the entire section. NuMA^+^ cells were counted in the acquired micrographs using ImageJ software cell counter plugin. The total area of sections with vessels and alveoli was determined using ImageJ software [18]. The number of NuMA^+^ cells per area was expressed as the cell number per 1 × 10^5^ µm^2^.

### Blood tests

After the experimental mice were euthanized, 1 mL of orbital sinus blood was collected. Liver function tests that measure alanine transaminase (ALT), aspartate transaminase (AST), alkaline phosphatase (ALP), glucose (Gluc), total protein (TP), total bilirubin (TB), and complete blood count were provided to assess liver damage.

### Statistical analysis

The data were analyzed using Statistica 13 and Microsoft Excel software. Factor analysis [19] of the obtained results was performed using factorial ANOVA or polychoric correlation for categorical variables. For variables where any factor effects were considered as significant (*p* < 0.05), the magnitude, direction and importance of the effects were assessed using Pareto charts. The impact of each factor on changes in variables was presented as statistical significance and absolute value of the standardized effect size (d). Additionally, the mean of the selected variables for each factor variation was presented as a bar graph to give a visual impression of the cell distribution and presence of complications. Correlations between important categorical variables were analyzed using Spearman’s rank correlation coefficient and were presented as ρ (degrees of freedom) = R Spearman statistic, statistical significance. The absolute value of correlation coefficient was interpreted as weak (< 0.2), sufficient (0.2–0.5), moderate (0.5–0.7), or strong (> 0.7).

## Results

### Characterization of isolated hAECs

**Fig. 3:**
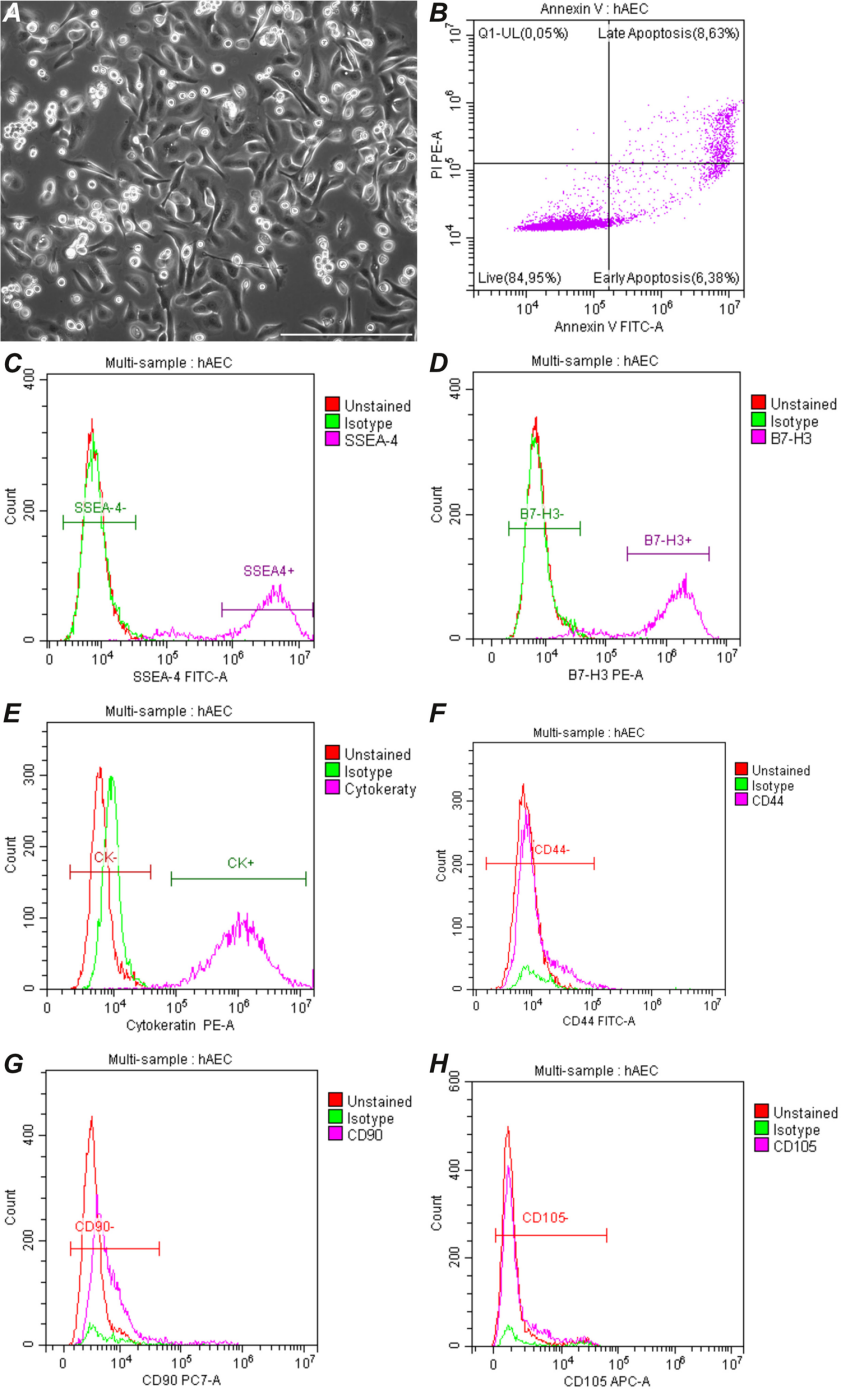
Morphological (**A**) and cytometric (B–H) characterization of isolated hAECs. After 24 h of culture, almost 80% of the isolated cells were viable, without apoptotic features (**B**). The cells identified in the isolated primary population included SSEA-4^+^ (**C**), B7H3^+^(**D**), CK14,15,16,19^+^(**E**), and a very small number of CD44^+^(**F**), CD90^+^(**G**), and CD105^+^(**H**)

After 24 h of culture, hAECs were characterized by fibroblast-like adherence and morphology. They expressed the immunomodulatory protein B7-H3 (99.71%), pluripotent cell marker SSEA-4 (99.57%), protein CD73 (95.89%), and epithelial cytokeratins 14,15,16, and 19 (87.50%). On the other hand, they minimally expressed mesenchymal cell markers, such as CD105 (5.32%), CD90 (1.69%), and CD44 (5.95%), which confirmed their epithelial nature (Fig. 3).

### The condition of recipient mice in the postoperative period

The general condition of the mice injected with cells directly into the spleen was good. One mouse died after direct intrasplenic administration in bolus of 2 × 10^6^ hAECs. The post-mortem examination could not conclusively determine the cause of death.

During the preparation of a subcutaneous splenic port, we did not observe complications such as hemorrhage or death of mice. During the 7-day observation period, there were no changes in animal behavior or postoperative wound complications. During and after the procedure of intrasplenic cell injection via the port, we found no deterioration in the general condition of any mice. These mice required only mild analgosedation, after which the postoperative recovery time was significantly shorter and animal mortality was lower compared to direct intrasplenic injection.

### Biochemistry and complete blood count of recipient mice

**Fig. 4:**
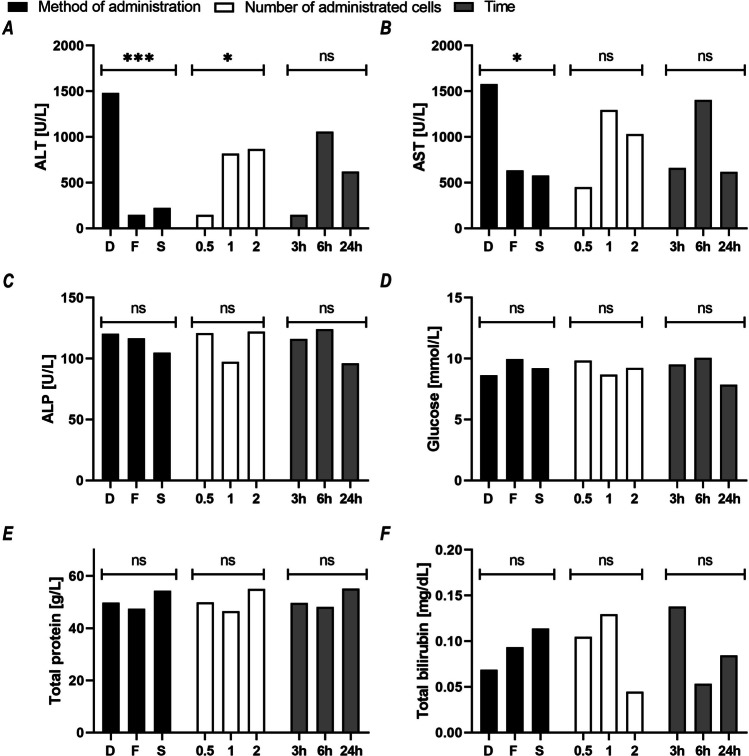
Assessment of markers of biochemical damage and function of the liver in recipient mice after hAEC administration. The graphs show the mean values for each variant of the studied factor: black = method of cell administration (D, direct; F, fast; S, slow), white = number of cells administered (0.5, 1, 2 × 10^6^), and gray = time elapsed from the cell administration to organ collection (3 h; 6 h; 24 h). For each variant of the studied factor, presented means were calculated from 27 mice representing 9 subgroups (method, number of cells, time; three mice each). *p < 0.05; ***p < 0.001 for the effect of a given factor on the change in the value of the variable under study; ns, statistically non-significant

The range of ALT and AST activity in the control group was 18–33 and 58–206.5 U/L, respectively. In all study groups, the activity of the tested aminotransferases was at least twice as high as in the control group. We observed a significant effect of the method of cell administration on the activities of ALT (*p* < 0.001; *d* = 0.57) and AST (*p* < 0.05; *d* = 2.94) and a significant effect of the cell dose (*p* < 0.05; *d* = 2.22) on the activity of ALT in the blood plasma of recipient mice. The activities of ALT and AST in mice receiving cells by direct injection were significantly higher than in animals receiving injections by other means. Both mice receiving 1 × 10^6^ and those receiving 2 × 10^6^ hAECs showed higher ALT activity than animals receiving the lowest cell dose (Fig. 4A, 4B). The method of administration, cell dose, and time of collection showed no significant effect on ALP activity or the concentrations of total protein, total bilirubin, and glucose, and their values did not differ significantly from the studied parameters in the control group (Fig. 4C-4F).

**Fig. 5:**
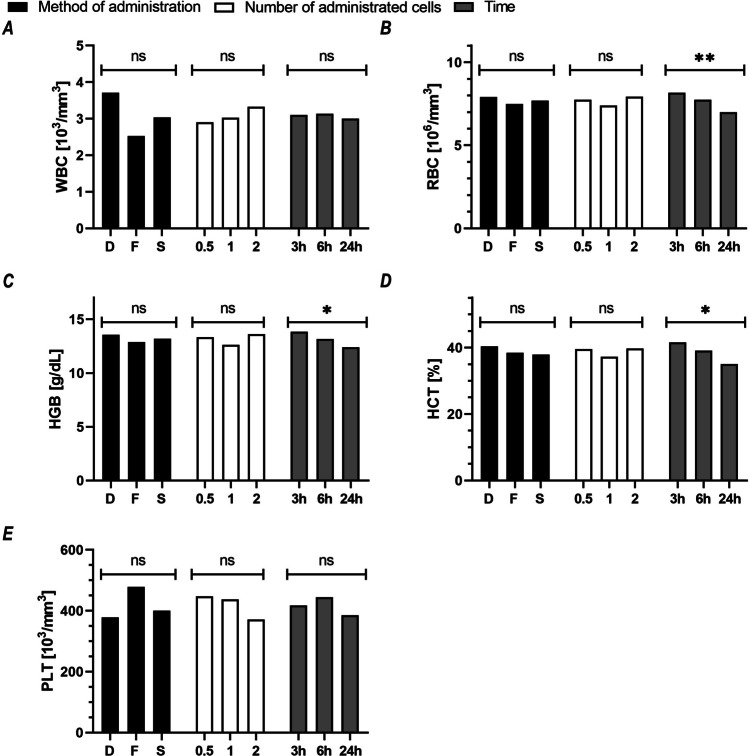
Assessment of complete blood count of recipient mice after hAEC administration. The graphs show the mean value for each variant of the studied factor: black = method of cell administration (D, direct; F, fast; S, slow), white = number of cells administered (0.5, 1, 2 × 10^6^), and gray = time elapsed from the cell administration to organ collection (3 h; 6 h; 24 h). For each variant of the studied factor, presented means were calculated from 27 mice representing 9 subgroups (method, number of cells, time; three mice each). *p < 0.05; **p < 0.01 for the effect of a given factor on the value change of the variable under study; ns, statistically non-significant

We found no significant effect of the administration method, cell dose, or collection time on most of the complete blood count parameters with the exception of a slight decrease in red blood cell parameters, not deviating from the normal ranges determined in the control group: RBC (*p* < 0.01; *d* = 2.85), HGB (*p* < 0.05; *d* = 2.42), HCT (*p* < 0.05; *d* = 3.24), depending on the time elapsed between cell administration and organ collection (Fig. 5).

### Histopathological assessment of the incidence of organ complications in recipient mice

#### Spleen

During the collection of organs at 3 h, 6 h, and 24 h after cell administration using all the described methods, we did not visualize the presence of hematomas or other splenic complications. During the necropsy of animals that had been fitted with a subcutaneous splenic port, we observed the formation of a thick fibrous capsule made of connective tissue. Microscopic examination of all spleens showed no lesions.

#### Liver

**Fig. 6:**
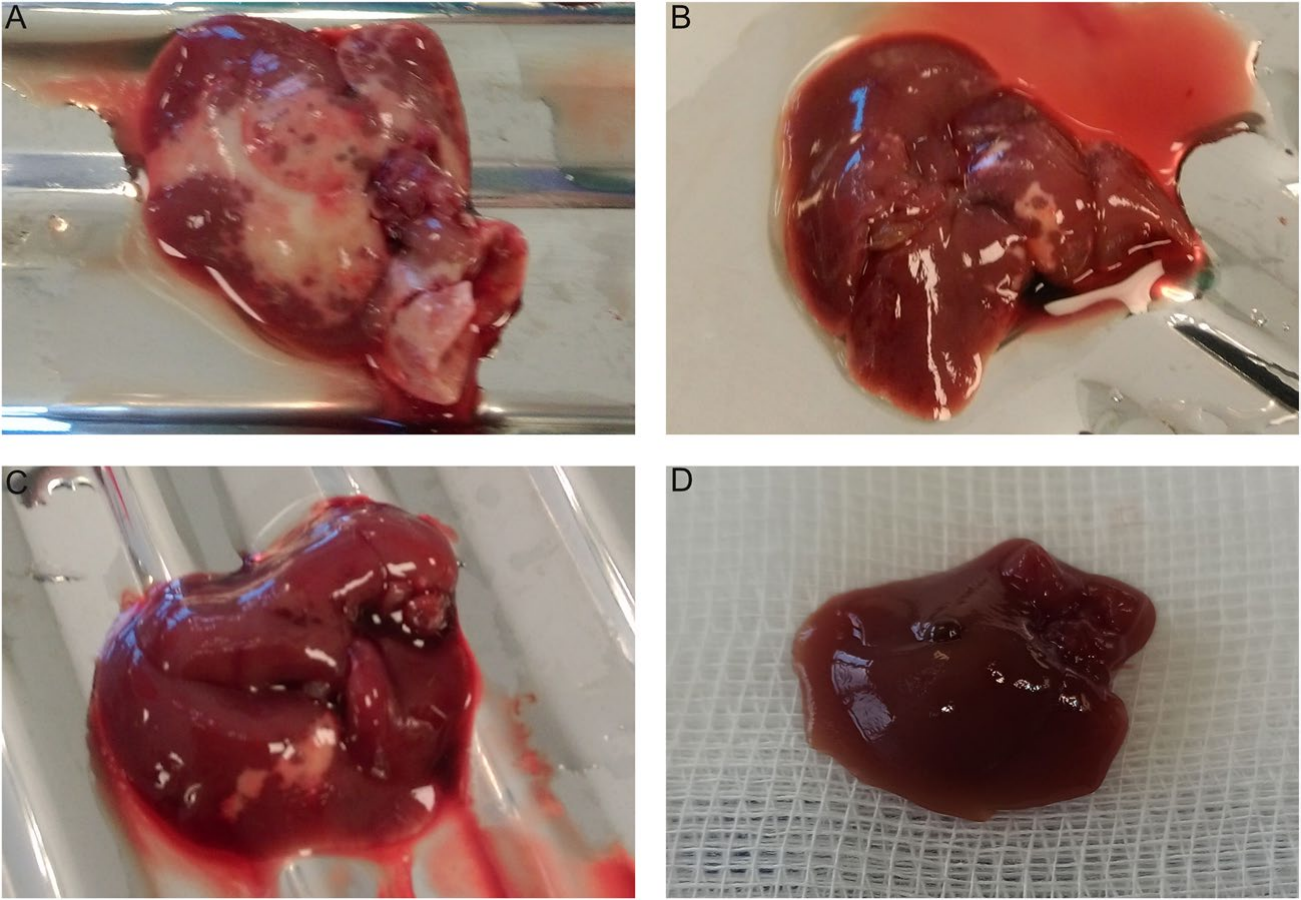
Macroscopic structure of the mouse liver 24 h after intrasplenic administration of human amniotic cells. The severity of lesions is visibly dependent on the administration method and number of hAECs. (**A**) after direct intrasplenic administration of 2 × 10^6^ hAECs; (**B**) after direct intrasplenic administration of 1 × 10^6^ hAECs; (**C**) after fast administration of 2 × 10^6^ hAECs via the splenic port; and (**D**) after slow administration of 1 × 10.^6^ hAECs via the splenic port. The livers of the mice representing this group showed no morphological differences from the livers of control mice (sham operation)

During necropsy, some of mice undergoing cell transplantation had white, confluent patches, which reflected the degree and extent of the histopathological changes in the organ and corresponded to ischemic lesions and necrotic areas on microscopic examination (Fig. 6).

Microscopic examination of control mice showed a normal histology of the liver. We found no inflammatory infiltrate, features of hepatocyte damage, or emboli in the vascular lumen (not shown).

**Fig. 7:**
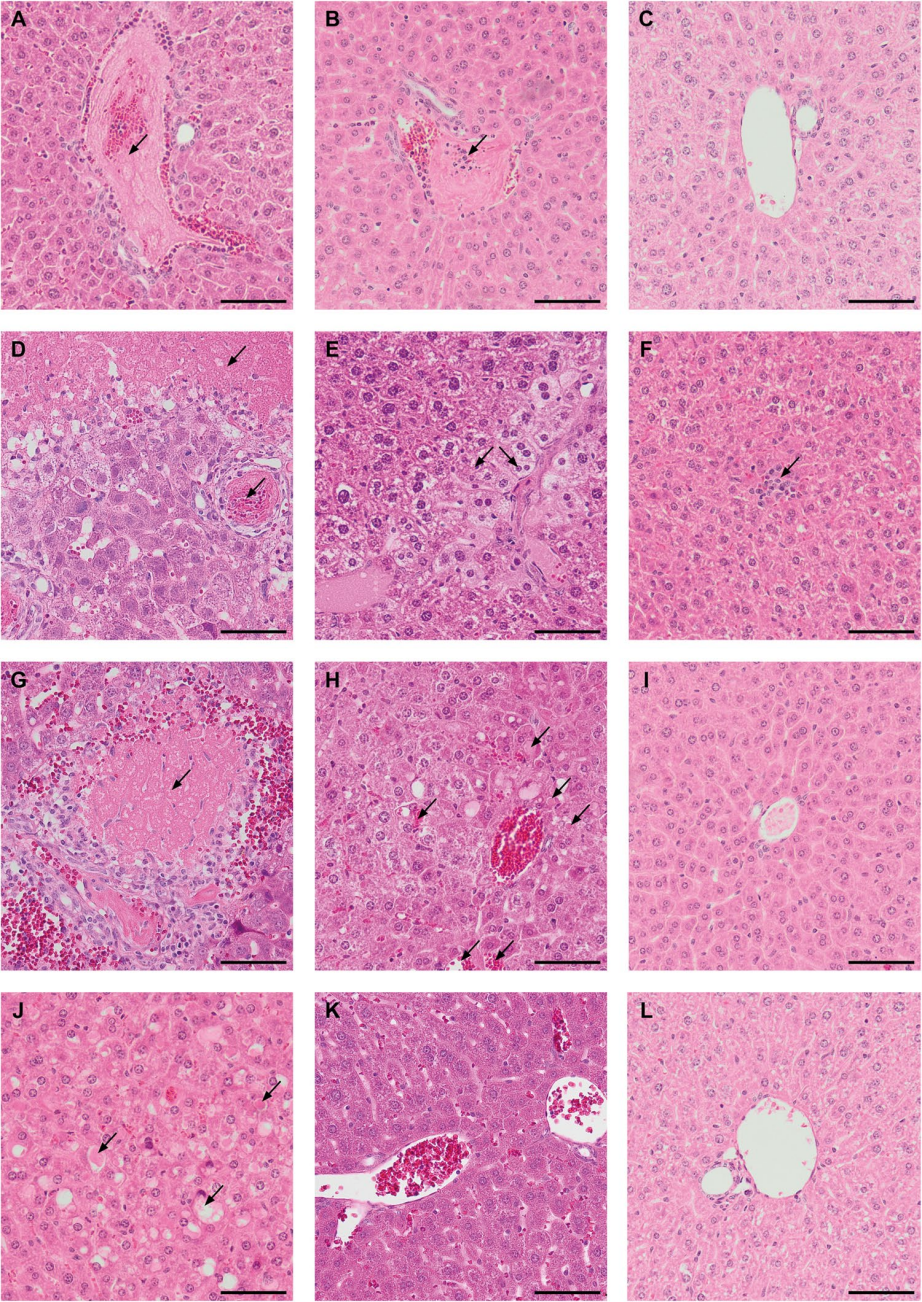
Histopathological changes in the liver observed after hAEC administration directly and via a subcutaneous splenic port. Examples of ischemic and embolic complications 6 h after: (**A**) direct administration of 2 × 10^6^ hAECs and (**B**) fast infusion of 0.5 × 10^6^ hAECs; as well as (**C**) lack of emboli after slow infusion of 0.5 × 10^6^ hAECs in veins. After direct intrasplenic administration of hAECs a massive necrosis of the liver parenchyma, characterized by a zonal pattern and embolic material in the form of an amyloid-like acidophilic mass in the interlobular veins were visible even at 24 h after administration of 2 × 10^6^ hAECs (**D**). Earlier, degenerated hepatocytes occupying zone 1 of the hepatic acinus were found at 6 h after administration of 1 × 10^6^ hAECs (**E**); and, at the same time, only single foci of inflammatory infiltrates in zone 2 were visible after administration of 0.5 × 10^6^ hAECs (**F**). Advanced histopathological changes were observed even within 24 h after fast infusion of 2 × 10^6^ hAECs via the splenic port, e.g. necrosis occurring around portal spaces and in the subcapsular area (**G**). The lesions were less pronounced after the administration of 1 × 10^6^ hAECs and were manifested at 6 h as dilated sinusoids and apoptotic eosinophilic bodies in zone 1 (**H**). At 6 h after administration of 0.5 × 10^6^ hAECs, there were no histological changes within liver acini (**I**). After 3 h of slow infusion of 2 × 10^6^ hAECs through the port, only one animal developed foci of swollen hepatocytes and numerous apoptotic bodies (**J**). At 24 h (**K**) and 6 h (**L**), structurally normal liver parenchyma with slight parenchymal edema or mild inflammation was observed after infusion of 1 × 10^6^ and 0.5 × 10^6^ cells, respectively. (A-L) H&E staining. The described histopathological changes are marked with arrows. Magn. × 200, scale bar represents 40 µm

We observed ischemic and embolic complications in the liver of recipient mice after intrasplenic implantation of hAECs, namely emboli in the lumens of small branches of the portal vein. These forms of an amyloid-like acidophilic mass, composed of formed elements of blood, fibrin, and administered cells were found mostly after direct administration and fast infusion hAECs (Fig. 7A and 7B), but not its slow infusion (Fig. 7C).

As early as 3 h after the intrasplenic administration of hAEC, histopathological changes were observed in the liver of mice, which intensified with the increase in the number of administered cells. After administration of 0.5 × 10^6^ hAECs, we observed in one mouse a small focus of subcapsular necrosis and one focus of severe hepatocyte degeneration around the central vein. After applying a dose of 1 × 10^6^ hAECs, we found the presence of emboli in the portal vessels, parenchymal edema, mild inflammation of periportal areas, and inflammatory foci in the parenchyma. After administration of 2 × 10^6^ hAECs, we observed in two mice foci of hepatocyte degeneration with numerous apoptotic bodies and profuse inflammatory infiltrate located within zone 1 of the hepatic acinus.

The use of higher doses of hAEC administered directly resulted in the occurrence of single or multiple foci of confluent necrosis and the presence of more extensive necrosis in livers collected 6 h and 24 h after administration of the cells, respectively, in the majority of mice (Fig. 7D, E). 24 h after administration of 2 × 10^6^ hAECs, a massive necrosis of the liver parenchyma, characterized by a zonal pattern; relatively spared areas of parenchyma located around the portal areas (zone 1 of the hepatic acinus); and embolic material in the form of an amyloid-like acidophilic mass in the interlobular veins, were visible. For comparison, at the same time points, administration of 0.5 × 10^6^ hAECs did not result in significant lesions apart from mild inflammation in the periportal areas (Fig. 7F).

A similar relationship was observed in mice that received fast hAEC infusion via the port. In livers collected 24 h after the administration of 0.5 × 10^6^ hAECs, we observed only mild inflammation within the portal areas, while after the application of the doses of 1 × 10^6^ and 2 × 10^6^, we observed moderate inflammation within the portal areas or the presence of foci of complete necrosis (Fig. 7G). In the earlier hours, histopathological changes were less severe (Fig. 7H, I). At 6 h and 3 h after administration of 0.5 × 10^6^, 1 × 10^6^, and 2 × 10^6^ hAECs, we observed mild or moderate inflammation and the presence of degeneration foci in the periportal areas. Only one mouse developed a single necrosis focus after administration of 2 × 10^6^ hAECs.

In comparison to the above mentioned methods of hAEC administration, after slow infusion of 0.5 × 10^6^, 1 × 10^6^, and 2 × 10^6^ hAECs via the port, we observed less advanced histopathological changes (Fig. 7J, K, L). After 3 h liver parenchyma showed normal structure or mild inflammation with slight parenchymal edema. Only one animal, administered with 2 × 10^6^ hAECs, developed emboli in the portal vessels and single foci corresponding to lesions with numerous eosinophilic bodies. The structure of the liver parenchyma remained normal even after 6 h regardless of the dose of cells used. Only one animal showed single foci of necrosis around some central veins. At 24 h after slow infusion of 0.5 × 10^6^, 1 × 10^6^, and 2 × 10^6^ hAECs, no significant lesions occurred apart from mild inflammation within the portal areas and hepatocyte edema in some animals.

**Fig. 8:**
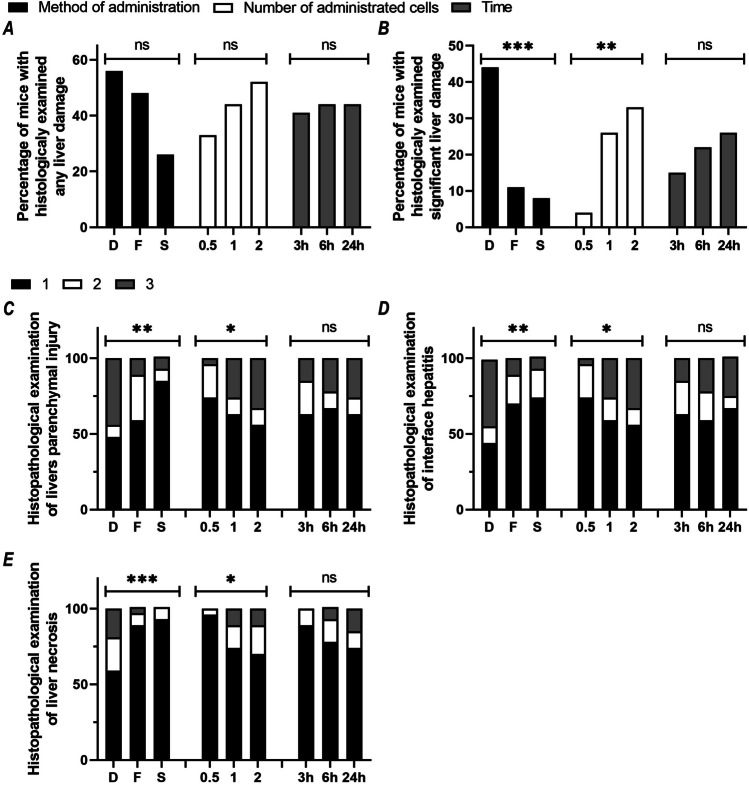
Histopathological assessment of liver damage. Percentages of mice meeting criteria for any (grade II) or significant (grade III) liver damage are shown on graph A and B, respectively. Percentages of mice scored as 1, 2, or 3 points on scales of histopathological markers of injury to the liver parenchyma, inflammation and necrosis are shown on graph C, D and E, respectively. For each variant of the studied factor, namely: method of cell administration (D, direct; F, fast; S, slow); number of cells administered (0.5, 1, 2 × 10^6^) and time elapsed from cell administration to organ collection (3 h; 6 h; 24 h), presented values were calculated from 27 mice. Statistically significant: *p < 0.05; **p < 0.01; ***p < 0.001; ns, statistically non-significant

We found no statistically significant effects of the method of administration, cell dose, or collection time on the percentage of mice showing at least minimal liver damage (grade II) on a semi-quantitative three-grade scale for histopathological assessment of inflammation, parenchymal injury, or necrosis (Fig. 8A). This dependence was manifested in almost twofold changes in the number of mice showing liver damage, namely its reduction after slow cell administration relative to direct and fast administration (*p* = 0.06; *d* = 1.95) and its increase after administration of high numbers of hAECs comparing to low numbers (*p* = 0.33; *d* = 1.47). However, the relationships between the method of administration or the number of administered cells and the percentage of mice showing advanced liver tissue damage (grade III) on histopathology were statistically significant (Fig. 8B). The percentage of mice with significant liver damage was highest in the groups after direct administration, and lowest after slow administration (*p* < 0.001; *d* = 3.50). The percentage of mice showing significant liver damage (grade III) increased with the number of administered cells (*p* < 0.01; *d* = 2.90) and cell infusion rate Table 5.

**Table 5:** The relationship between the number of cells administered per unit of time and the significant liver damage (grade III). The groups of mice (1)-(9) differed in the number of administered hAECs (0.5 mln, 1 mln, 2 mln) suspended each time in 250 µl of 0.9% NaCl, and the rate of their infusion (10 µl/min, 20 µl/min, 400 µl/min)

	Injected cell number					
	0.5 million (2 × 10^6^ / ml)		1 million (4 × 10^6^ / ml)		2 million (8 × 10^6^ / ml)	
Infusion rate	Number of cells administered per unit of time	Incidence of significant liver damage	Number of cells administered per unit of time	Incidence of significant liver damage	Number of cells administered per unit of time	Incidence of significant liver damage
400 µl/min	(1) 0.8 × 10^6^ / min	11%	(2) 1.6 × 10^6^ / min	56%	(3) 3.2 × 10^6^ / min	67%
20 µl/min	(4) 0.04 × 10^6^ / min	0%	(5) 0.08 × 10^6^ / min	11%	(6) 0.16 × 10^6^ / min	22%
10 µl/min	(7) 0.02 × 10^6^ / min	0%	(8) 0.04 × 10^6^ / min	11%	(9) 0.08 × 10^6^ / min	11%

We observed a significant effect of the method of hAEC administration on the results of histopathological analysis performed using the scoring scales assessing hepatitis (*p* < 0.01; *d* = 2.93), parenchymal injury (*p* < 0.01; *d* = 3.37), and severity of hepatic necrosis (*p* < 0.001; *d* = 3.77). We found a statistically significant effect of the number of administered cells on the distribution of scores in histopathological scales for grading hepatitis (*p* < 0.05; *d* = 2.21), parenchymal injury (*p* < 0.05; *d* = 2.10), and severity of hepatic necrosis (*p* < 0.05; *d* = 2.59). We found no relationship between the time that elapsed from cell administration to organ collection and the score distribution of the examined histopathological parameters used for grading hepatitis (*p* = 0.83; *d* = 0.20), parenchymal injury (*p* = 0.86; *d* = 0.17), and severity of hepatic necrosis (*p* = 0.09; *d* = 1.71) (Fig. 8C-E).

#### Lungs

The lack of visible respiratory signs and good general condition of the animals after cell transplantation indicated the absence of pulmonary complications in the applied models of cell administration. On microscopic examination, we found in all mice normal lung parenchyma, which did not differ from that of the control group (not shown).

### Distribution of transplanted hAECs in organs of recipient mice

**Fig. 9:**
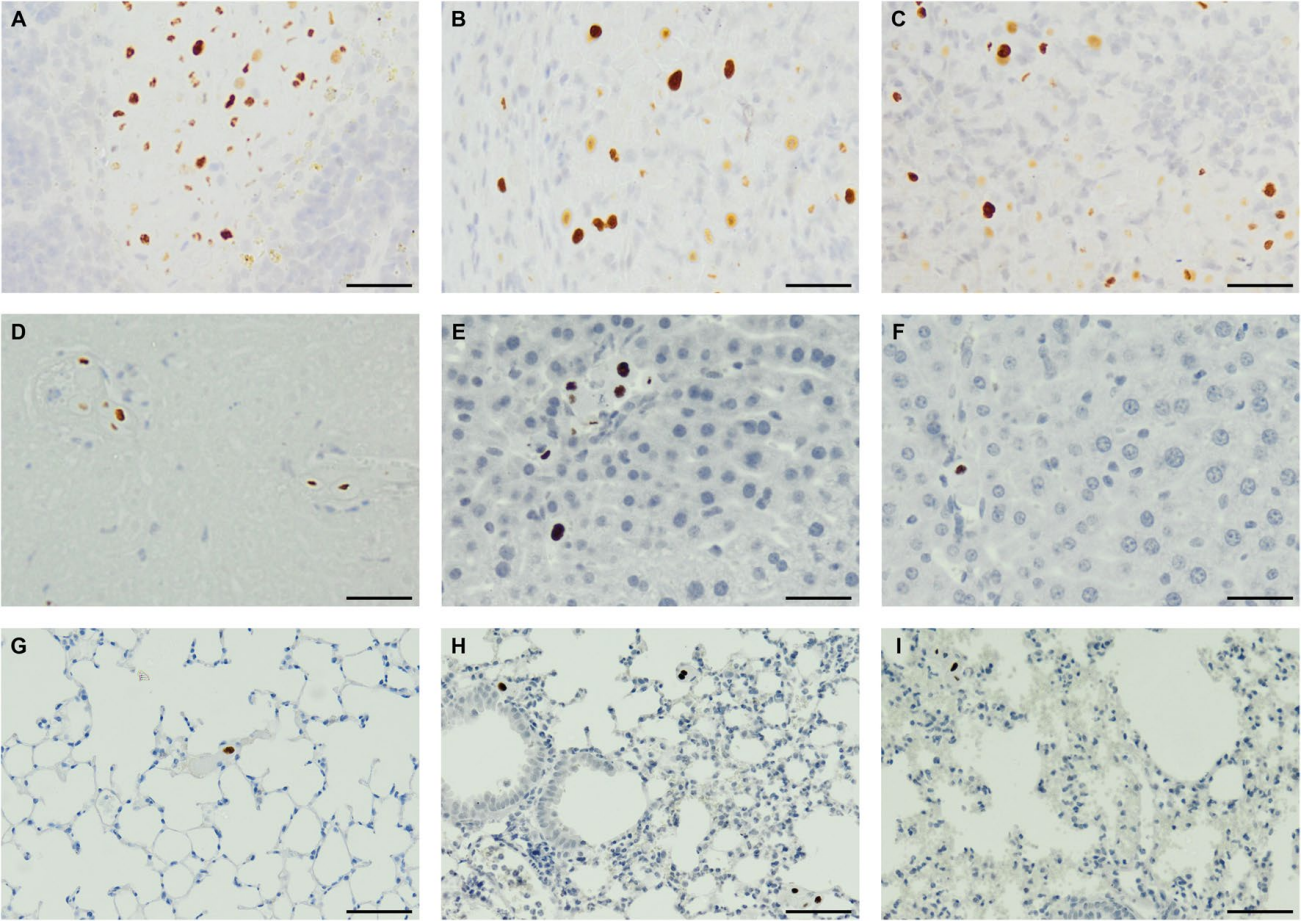
Immunodetection of NuMA^+^ hAECs in the spleen (A, B, C), liver (D, E, F) and lungs (G, H, I) 6 h after administration of 2 × 10^6^ hAEC. (A, D, G): direct intrasplenic bolus injection,; (B, E, H): intrasplenic port—fast infusion; (C, F, I): intrasplenic port—slow infusion. Mag. × 200, scale bar: 40 µm

Depending on the method of administration, we found the presence of NUMA^+^ hAECs in the splenic parenchyma in approximately 20–70% of recipient mice. These cells were usually present at the injection site in the form of relatively large clusters. Most numerous hAEC NuMA + cells were visualized 6 h after direct cell administration. We did not observe any changes in their distribution within the splenic parenchyma during the experiment (Fig. 9A-C). Intriguingly, despite the immunohistochemical confirmation of hAEC presence in the spleen, we did not find any hAECs in the liver parenchyma of mice without thromboembolic complications. In the livers of mice with such complications (those after direct infusion), the number of hAECs was relatively highest, and these cells were localized within the lumen of interlobular veins and nearby portal branches and necrotic areas (Fig. 9D-F). In some mice, single NuMA^+^ cells were distributed in the lumen of lung capillaries (Fig. 9G-I).

### Analysis of the effect of the method of hAEC administration, number of administered hAECs, and time elapsed since their administration on distribution of hAECs in the organs of the recipient mice

**Fig. 10:**
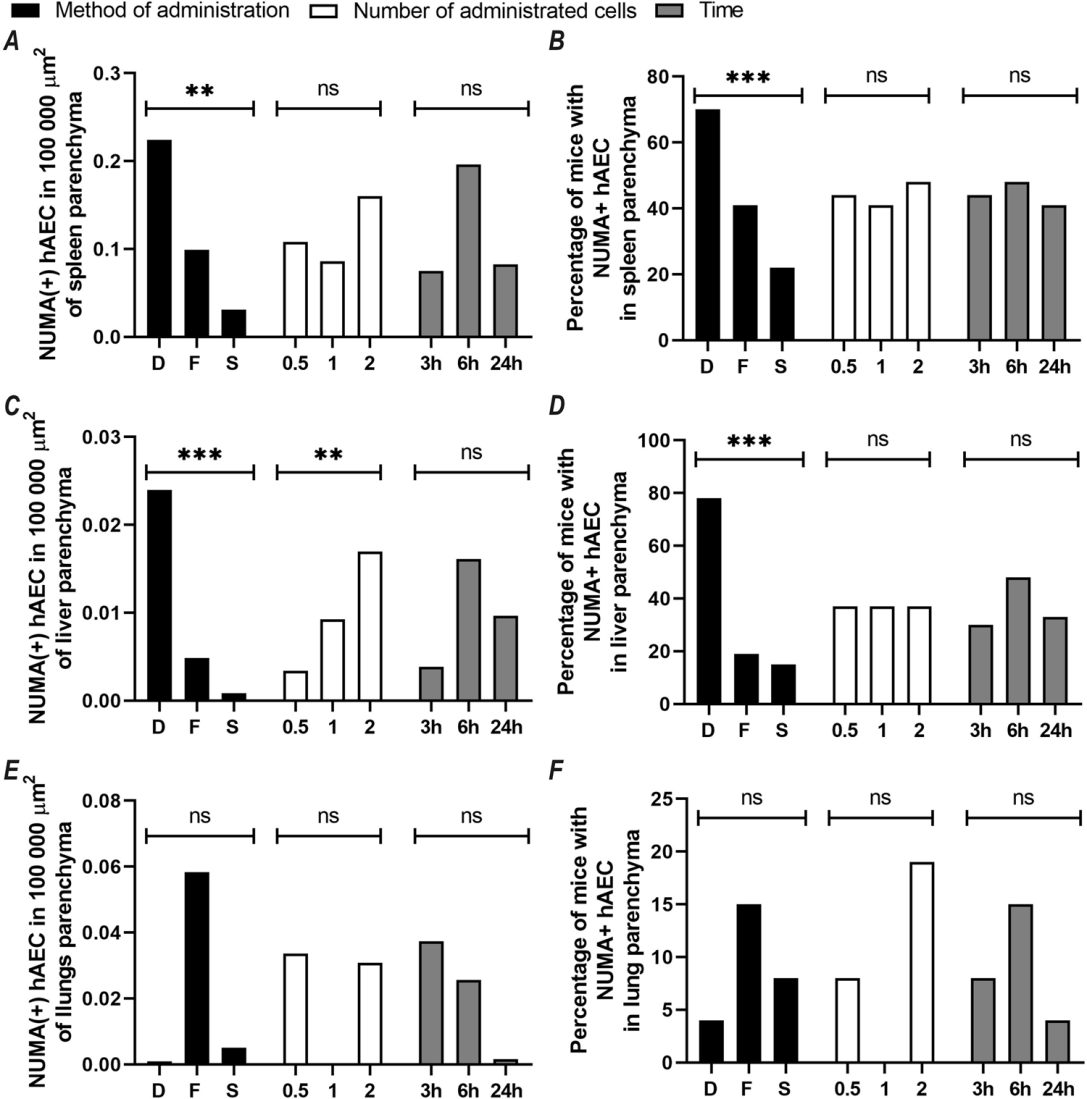
The effect of the method of hAEC administration, number of administered hAECs, and time elapsed since they were given on distribution of hAECs in the spleen, liver, and lungs of recipient mice. The graphs on the left show the number of NuMA^+^ hAECs per 10^5^ µm^2^ area; the graphs on the right show the percentage of mice with NuMA^+^ hAECs in the studied tissues. The graphs show the mean value for each variant of the studied factor: black = method of cell administration (D, direct; F, fast; S, slow), white = number of cells administered (0.5, 1, 2 × 10^6^), and gray = time elapsed from the cell administration to organ collection (3 h; 6 h; 24 h). For each variant of the studied factor, presented values were calculated from 27 mice. Statistically significant: *p < 0.05; **p < 0.01; ***p < 0.001; ns, statistically non-significant

We noted a significant effect of the method of hAEC administration on the number of implanted NUMA^+^ hAECs (*p* < 0.01; *d* = 3.32) and the percentage of mice showing NUMA^+^ hAECs in the splenic parenchyma (*p* < 0.001; *d* = 4.16). As the infusion rate increased, we observed a significant increase in the number of NUMA^+^ cells and the percentage of mice showing NuMA^+^ cells in the splenic parenchyma (Fig. 10A, B).

We showed a significant effect of the method of hAEC administration on the number of NUMA^+^ cells in the liver parenchyma (*p* < 0.001; *d* = 4.47) and the percentage of mice showing NUMA^+^ hAECs in the liver parenchyma (*p* < 0.001; *d* = 5.81) (Fig. 10C, D; Table 6). We observed the highest number of NuMA^+^ cells in the liver parenchyma in the groups with direct intrasplenic administration and the lowest number in the groups with slow administration via the splenic port. The effectiveness of hAEC implantation in the liver parenchyma was significantly influenced by the number of administered cells (*p* < 0.01; *d* = 2.66). The number of NUMA^+^ hAECs implanted in the liver parenchyma increased with the number of administered cells, but the percentage of mice showing NUMA^+^ hAECs in the liver parenchyma (*p* = 0.98; *d* = 0.04) did not increase during this time. In addition, the method of cell administration analyzed together with their number administered to mice had a significant effect on the number of NUMA^+^ hAECs in the liver parenchyma (*p* < 0.05; *d* = 2.51). In contrast, we found no effect of the time of liver collection on the number of NUMA^+^ hAECs present in the parenchyma (*p* = 0.07; *d* = 1.81) and the percentage of mice showing NUMA^+^ hAECs in the parenchyma (*p* = 0.19; *d* = 1.32).

**Table 6:** The relationship between the number of cells administered per unit of time and the percentage of mice showing the presence of transplanted hAECs in the liver parenchyma. The groups of mice (1)-(9) differed in the number of administered hAECs (0.5 mln, 1 mln, 2 mln) suspended each time in 250 µl of 0.9% NaCl, and the rate of their infusion (10 µl/min, 20 µl/min, 400 µl/min)

	Injected cell number					
	0.5 mln (2 × 10^6^ / ml)		1 mln (4 × 10^6^/ml)		2 mln (8 × 10^6^/ml)	
Infusion rate	Cells administered per unit of time	Percentage of mice showing transplanted hAECs	Cells administered per unit of time	Percentage of mice showing transplanted hAECs	Cells administered per unit of time	Percentage of mice showing transplanted hAECs
400 µl/min	(1) 0.8 × 10^6^/min	78%	(2) 1.6 × 10^6^/min	78%	(3) 3.2 × 10^6^/min	78%
20 µl/min	(4) 0.04 × 10^6^/min	22%	(5) 0.08 × 10^6^/min	11%	(6) 0.16 × 10^6^/min	22%
10 µl/min	(7) 0.02 × 10^6^/min	11%	(8) 0.04 × 10^6^/min	22%	(9) 0.08 × 10^6^/min	11%

We did not observe a significant effect of the administration method, cell dose, and collection time either on the number of implanted NuMA^+^ hAECs or the percentage of mice showing NuMA^+^ hAECs in the lung parenchyma (Fig. 10E, F). Nevertheless, a number of hAEC after fast administration via the port was more elevated in lungs, as compared to other technics of infusion.

We observed a significant positive correlation between the percentage of mice showing NUMA^+^ hAECs in the splenic parenchyma and the percentage of mice showing NUMA^+^ hAECs in the liver parenchyma (ρ(79) = 0.34, *p* < 0.01). A similar correlation was not found when comparing the percentage of mice showing NUMA^+^ hAECs in the lung, liver (ρ(79) = -0.05, *p* = 0.63) and spleen (ρ(79) = -0.10, *p* = 0.38). A sufficient positive correlation between the percentage of mice showing NUMA^+^ hAECs in the liver and a significant liver damage (III) on histopathology was statistically significant (ρ(79) = 0.23, *p* < 0.05). A strong positive correlation was found between the percentage of mice showing NUMA^+^ hAECs in the liver and the severity of inflammatory response on histopathology (ρ(79) = 0.27, *p* < 0.05). The weak correlation between the presence of NUMA^+^ hAECs in the liver, and the severity of parenchymal injury (ρ(79) = 0.16, *p* = 0.15), on the one hand, and severity of necrosis on histopathology (ρ(79) = 0.20, *p* = 0.07), on the other, was not statistically significant (Table 7).

**Table 7:** Correlations between variables determining the presence of administered NuMA^+^ hAECs in the parenchyma of the examined organs and the presence of complications in the liver parenchyma. Correlations are presented as ρ (degrees of freedom) = R Spearman statistic, statistical significance

	% of mice with NuMA^+^ cells in spleen	% of mice with NuMA^+^ cells in liver	% of mice with NuMA^+^ cells in lung
% of mice with NuMA^+^ cells in liver	**Ρ(79) = 0.34, *p* = 0.0017**	-	-
% of mice with NuMA^+^ cells in lung	Ρ(79) = -0.10, *p* = 0.38	ρ(79) = -0.05, *p* = 0.63	-
% of mice with any liver damage (II)	ρ(79) = -0.07, *p* = 0.49	ρ(79) = 0.15, *p* = 0.16	ρ(79) = 0.17, *p* = 0.11
% of mice with significant liver damage (III)	ρ(79) = -0.03, *p* = 0.76	**ρ(79) = 0.23, *p* = 0.036**	ρ(79) = 0.05, *p* = 0.61
Histopathological examination of interface hepatitis	ρ(79) = -0.02, *p* = 0.83	**ρ(79) = 0.27, *p* = 0.015**	ρ(79) = 0.11, *p* = 0.30
Histopathological examination of parenchymal injury	ρ(79) = -0.08, *p* = 0.45	ρ(79) = 0.16, *p* = 0.15	ρ(79) = 0.12, *p* = 0.28
Histopathological examination of necrosis	ρ(79) = -0.06, *p* = 0.60	ρ(79) = 0.20, *p* = 0.07	ρ(79) = 0.05, *p* = 0.65

## Discussion

A therapy using systemically administered cell suspension, like any other medical procedure, carries a risk of complications. So far, adverse reactions after systemic cell delivery have been observed in animal models [20], during clinical experiments [21] and unapproved cell therapies [22]. The most commonly observed complications of cell therapies include adverse immune reactions [23], infectious complications [24], neoplastic transformation of the administered cells [25], and formation of vascular emboli [26].

The aim of this experiment was to optimize the delivery of stem cells isolated from the human amnion, applied as part of experimental cell therapy for liver diseases, which would enable effective and side-effect-free distribution of transplanted cells in the liver of the recipient. We assessed how the technique of administration, number of administered cells, and time after administration affect the cell distribution and the occurrence of histopathological changes. We compared three techniques of stem cell administration: direct intrasplenic administration and administration via a splenic port—fast and slow.

We prepared a subcutaneous splenic port in mice according to the procedure described by Miki et. al. [12]. Compared to direct intrasplenic administration, where the integuments are incised to make cell delivery quick and efficient, the use of the model of subcutaneous port administration allowed for significant prolongation of the cell suspension infusion. Due to the fact that the administration of cells in this model involves only insertion of a needle into a palpable splenic port, the animals required only mild analgosedation, after which the postoperative recovery time was significantly shorter and animal mortality was lower compared to direct intrasplenic injection. After the injection of cells via the port, we did not observe any alarming signs and the observed decrease in red blood cell parameters did not significantly affect the condition of the animals.

The obtained results indicate that the incidence and severity of markers of liver damage depend both on the number of administered cells and the rate of their infusion. In some mice, after the administration of amniotic cells, we observed changes corresponding to ischemic injury to the liver parenchyma caused by the presence of visible microemboli in the portal system. In clinical practice, similar morphological and pathophysiological alterations may occur during celiac trunk thrombosis [27] and intravenous delivery of hematopoietic cells [28]. We demonstrated that as the number of administered cells increases, so does the incidence of histopathological injury to the liver parenchyma, including embolic complications. The observed positive relationship between the number of administered hAECs and the incidence of complications is most likely due to the number of cells administered per unit of time. Regardless of the administered dose, the cells were each time suspended in 250 µl of normal saline. Therefore, the study groups differed in the number of administered cells and their dilution (0.5 million: 2 × 10^6^/ml; 1 million: 4 × 10^6^/ml; 2 million: 8 × 10^6^/ml). After infusion of 0.5 million hAECs, serious embolic complications were observed in only 4% of the animals. In contrast, after the administration of 1 and 2 million hAECs, the incidence of serious embolic complications was 26% and 33%, respectively. We demonstrated that the number of administered cells does not affect the percentage of animals with hAECs identified in the liver, spleen, and lung.

The number of serious embolic complications also increased proportionally with the cell injection rate. After direct intrasplenic administration of 250 µl of cell suspension over approximately 30 s (400 µl/min), the incidence of serious embolic complications was as high as 44%. The use of fast (20 µl/min) and slow infusion (10 µl/min) via the splenic port allowed us to reduce the incidence of serious complications to 11% and 7%, respectively. Interestingly, as the infusion rate decreased, the effectiveness of intrasplenic administration also decreased significantly. When using fast and slow infusion via the port, we observed a significant decrease in the percentage of mice showing hAECs in the liver parenchyma compared to the animals directly injected (in fast mode) with cell suspension. Our results indicate that a slower administration of a smaller number of cells via the intrasplenic route allows for a significant improvement in the safety of the therapy used but it may also potentially reduce its effectiveness due to worse distribution in the liver. The use of a slower infusion probably allowed even distribution of the implanted cells in the parenchyma of the spleen, which acted as a buffer gradually releasing the administered cells into the confluence of the portal vein. An alternative hypothesis for the lower complication rate with slower infusion is that the administered cells immediately enter the splenic vascular area. In this case, the absence of emboli would be due to a much lower cell flow through the portal system per unit of time. Both presented hypotheses regarding the lower incidence of embolic complications when using slow cell infusion seem equally plausible.

One of the many requirements for introducing experimental cell therapy to the stage of clinical studies and then clinical practice is to understand the distribution of administered cells and the mechanisms driving this process. The administered cells may show inadequate distribution in the target organ or may be located in other organs, increasing the risk of therapy complications. The distribution of cells in the tissues is not uniform and is highly dependent on the route of administration and the pathological process taking place in the body [7, 10]. After systemic delivery of cell suspension, during cell homing, passive and active mechanisms work to varying degrees, leading to the entrapment of the administered cells in the vascular network and their transmigration through the vascular endothelium. The active mechanism concerns cells capable of migration and diapedesis; it occurs mainly in postcapillaries, where the relatively wide diameter of the vessels and the slow-flowing blood current create favorable conditions for this process to occur. The passive cell homing mechanism occurs in capillaries whose diameter is usually smaller than the diameter of the administered cells. When passing through the vascular network of a given organ, the cells become congested in the lumen of the vessel. The resulting microembolism causes ischemia of the vessel wall, which results in endothelial gaping. Subsequently, molecular mechanisms, together with the force of the flowing blood current, lead to transmigration of cells and their settlement in the parenchyma of the organ [29]. Our data indicate that in the case of hAECs administered intrasplenically via the port and using direct injection, cell homing is mainly the responsibility of the passive mechanism. We observed that hAECs were unevenly distributed in the liver parenchyma, mainly in the interlobular veins as well as in their close proximity (sinusoids in zone 1 of the hepatic acinus), i.e. in the terminal branches of the portal vein. On histopathology, we observed emboli in the lumens of small branches of the portal vein, composed of formed elements of blood, fibrin, and administered cells. The diameter of hepatic sinusoids in mice and humans is similar (5–15 µm) [30–32] and much smaller than the diameter of administered hAECs (15–25 µm). This largely explains why we did not observe the administered cells in the central veins. Also, zonation of parenchymal injury consisting in the occurrence of necrosis, eosinophilic bodies, and hepatocyte degeneration within zone 1 of the hepatic acinus suggests the cellular mechanism of injury. Only a small portion of animals also exhibited the presence of embolic material within the relatively large branches of the portal vein. The formation of emboli within larger vessels can be explained by the fact that every cell and tissue apart from vascular endothelium has some thrombogenic properties. In addition, due to their viscosity, nucleic acids escaping from dead cells contribute to the formation of cellular aggregates that may obstruct larger vessels [33]. Similar observations were made by Timm and Vollmar [34] after the administration of hepatocytes in rats, who with the help of intravital fluorescence microscopy observed the formation of microemboli within the small branches of the portal vein and obtained comparable data relating to the localization of the administered cells in the liver parenchyma.

Our data show that the distribution effectiveness of the administered amniotic cells, measures as the percentage of animals showing hAECs in the liver parenchyma, does not depend on the number of administered cells but only on the method of their administration and the infusion rate. The implantation efficacy using direct administration (400 µl/min) was high and reached 78%. In contrast, the use of infusion via the port (20 µl/min and 10 µl/min for fast and low infusion, respectively) resulted in low implantation effectiveness ranging from 11 to 22%. The observed correlation between serious embolic complications and high effectiveness of distribution in the liver is most likely related to the passive mechanism of cell homing, where the administered cells formed emboli that induced vascular endothelial necrosis and created favorable hemodynamic conditions for passage of cells into the organ parenchyma.

Within the examined time interval after the administration of cells (3–24 h), we did not observe any significant differences in the distribution of cells within the spleen, liver, and lungs. Regardless of the injection rate and the number of administered cells, hAECs were localized mainly in the splenic parenchyma. In contrast, the number of cells per comparable area of the liver section was approximately ten times lower than observed in the spleen. Taking into account spleen and liver volumes in mice, which are 0.18 ml and 1.65 ml, respectively, it can be estimated that the number of hAECs in both organs was very similar. Similar observations were made by Miki et. al., who after intrasplenic administration via the port noted that the majority of hAECs was localized in equal proportions in the spleen and liver. Similarly to our study, these authors obtained low effectiveness of cell distribution in the lungs [12]. Different results were obtained by Srinivasan et. al., who after intrasplenic administration of hAECs observed them mainly in the liver parenchyma [10].

Our data confirm significant contribution of the passive cell homing mechanism, in which the disproportion between the diameter of administered cells and the diameter of the microcirculatory vessels induced the formation of a microemboli from the administered cells. It seems that this is a prerequisite for the occurrence of favorable hemodynamic conditions enabling the passage of the administered cells from the vascular area to the organ parenchyma. In contrast to the organs characterized by the so-called ‘end-circulation’, such as the heart and brain, the liver parenchyma, thanks to its double vascularization through the arteries extending from the celiac trunk and the venous portal system, shows a certain resistance to ischemic complications during the formation of emboli in microcirculation. When delivering cells to the spleen or the portal system, the administered cell number and infusion rate should be rationally balanced so that the scale of microemboli formation in the microcirculation ensures satisfactory distribution in the liver without significant impairment of the function and morphology of the liver parenchyma.

To sum up, some previous studies indicated that achieving the proper concentration of a therapeutic agent in damaged tissue is the cornerstone of therapeutic success and significantly reduces the occurrence of adverse effects. To achieve the most efficient distribution of stem cells, the most effective routes of administration are sought [7]. Both preclinical and clinical trials have demonstrated that direct administration of cells or therapeutic substances intrahepatically, or intrasplenically results in high substance distribution in the liver and reduces systemic distribution of the therapeutic agent [35, 36]. It has been repeatedly shown that drug administration or stem cell transplantation into the spleen in humans can be both effective and safe [37]. Unfortunately, intrasplenic administration, especially repeated, in animal models poses significant technical challenges. While direct intrasplennic administration is possible in humans and larger animals, such as under USG guidance, in smaller animals like rodents, the creation of a splenic port is necessary, which provides a safe alternative to multiple surgeries required for repeated cell administration in a mouse model [12].

In this study we proved the usefulness of the subcutaneous splenic port, which allowed us to significantly extend the infusion time of the cell suspension and thus to reduce the number of complications of human cell transplantation in a mouse model of experimental cell therapy. Reducing the number of administered cells allowed for a significant reduction of complications and did not adversely affect the effectiveness of cell distribution in the liver. On the other hand, reducing the rate of infusion significantly improved the safety profile of the performed procedure but at the cost of reduced effectiveness. The observed damage and the relationship between the tested factors and the distribution of the transplanted human amniotic cells in the mouse liver suggests a predominant involvement of a passive cell homing mechanism, in which emerging microemboli induce the passage of the administered cells to the liver parenchyma. Due to the invasive nature of the procedure of intrasplenic delivery, both in direct injection and via the port, and a certain percentage of complications, when selecting the method of administration, the potential benefits should be weighed against the risks that may be posed by cell therapy. The choice of the appropriate delivery technique and dose of implanted cells should depend on the nature and course of the animal model of pathology that we are trying to treat with cell therapy. In models of liver damage, where the final survivability of animals depends on the rapid control of inflammation and restoration of the organ function and thus obtaining the correct values of critical parameters, we suggest considering the delivery of 1 × 10^6^ cells by slow infusion via the port. The use of this method of cell administration will significantly reduce the incidence of complications that could result from the therapeutic procedure technique itself and significantly worsen the condition of animals and the outcome of treatment at the expense of a slight decrease in the effectiveness of distribution in the liver. The data obtained will allow for a better understanding of the mechanisms responsible for cell homing and organ distribution of cells, which will translate into greater effectiveness of preclinical experimental cell therapies for liver diseases and our conclusions will help to design safer and more effective cell therapies.

## Data Availability

The data presented in this study are available in the article and will be made available on reasonable request.
